# Fahr Syndrome Identified During Evaluation of Recurrent Seizure-Like Activity in a Middle-Aged Woman

**DOI:** 10.7759/cureus.116602

**Published:** 2026-09-20

**Authors:** Travis Sekhon, Elias Malouf

**Affiliations:** 1 Department of Internal Medicine, McLaren Macomb Hospital, Mount Clemens, USA; 2 Department of Internal Medicine, Oakland University, Rochester, USA

**Keywords:** basal ganglia calcification, encephalopathy, fahr syndrome, intracranial calcification, seizures

## Abstract

Fahr syndrome is a rare neurologic disorder characterized by bilateral intracranial calcifications, most commonly involving the basal ganglia, and is typically diagnosed after exclusion of secondary metabolic, infectious, and endocrine causes. Clinical manifestations are variable and may include seizures, cognitive impairment, behavioral changes, movement abnormalities, and altered mental status. We present the case of a 55-year-old woman with recurrent episodes of altered mental status and seizure-like activity whose neuroimaging consistently demonstrated extensive, symmetric cerebral and cerebellar calcifications. Despite multiple hospitalizations and a comprehensive diagnostic evaluation, including metabolic, infectious, and structural workup, no alternative etiology was identified. The patient was ultimately diagnosed with Fahr syndrome. This case highlights the importance of recognizing Fahr syndrome in patients with characteristic imaging findings during the evaluation of common neurologic presentations and emphasizes the role of a systematic, exclusion-based approach to diagnosis.

## Introduction

Fahr syndrome is a rare neurologic disorder characterized by bilateral intracranial calcifications, most commonly involving the basal ganglia, thalami, dentate nuclei, and cerebral cortex [1,2,3]. Although some patients remain asymptomatic, Fahr syndrome can present with a broad spectrum of neurologic and neuropsychiatric manifestations, including movement disorders, seizures, cognitive impairment or dementia, speech impairment, oculomotor abnormalities, choreoathetosis, and ataxia, with symptoms most commonly emerging during the fourth or fifth decade of life [3,4,5]. Intracranial calcifications may occur as a primary idiopathic or familial disorder, often termed Fahr disease, or may occur secondary to metabolic, endocrine, infectious, or toxic etiologies, in which case the condition is termed Fahr syndrome [1,4,6,7]. Given this variable presentation, intracranial calcifications are often discovered incidentally during neuroimaging performed for more common neurologic complaints, making clinical correlation and evaluation for secondary causes essential [1,4,8].

Diagnosis relies on neuroimaging findings in combination with clinical manifestations and evaluation for potential secondary causes, including metabolic, infectious, toxic, traumatic, or structural etiologies [4]. Dysregulation of calcium-phosphate metabolism plays a central role in secondary Fahr syndrome. In hypoparathyroidism, low parathyroid hormone levels impair calcium and phosphate homeostasis, leading to hypocalcemia and hyperphosphatemia and promoting abnormal calcium-phosphate deposition within intracranial structures [1,4]. This case highlights recurrent neurologic symptoms in the setting of bilateral intracranial calcifications and chronic calcium-phosphate abnormalities, ultimately supporting a diagnosis of secondary Fahr syndrome.

## Case presentation

A 55-year-old female with a past medical history of hypothyroidism, chronic kidney disease, chronic hypocalcemia, migraine disorder, chronic pain, alcohol use disorder, and squamous cell carcinoma of the upper esophagus status post chemotherapy and radiation therapy presented to the ED with recurrent episodes of altered mental status and seizure-like activity over the preceding year.

The patient initially presented to the ED in April 2023 for evaluation of confusion and atypical twitching reported by her husband. CT imaging at that time revealed extensive bilateral cerebral and cerebellar calcifications (Figure 1). The patient's symptoms improved throughout the hospitalization, and she elected to leave against medical advice before a complete diagnostic workup could be performed.

**Figure 1:**
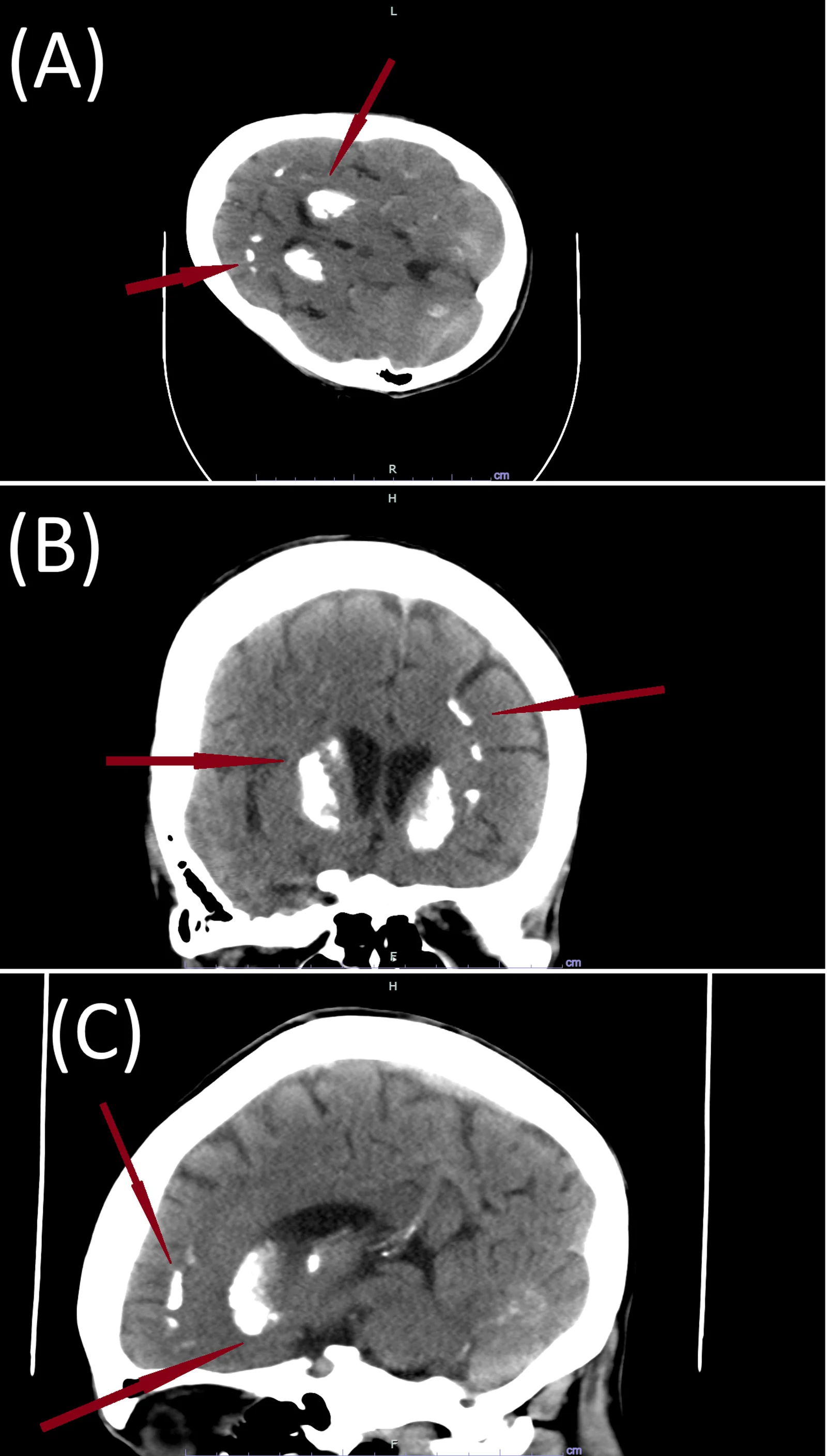
Noncontrast computed tomography of the head demonstrating extensive bilateral intracranial calcifications. Axial (A), coronal (B), and sagittal (C) noncontrast computed tomography images of the head demonstrate dense, symmetric intracranial calcifications involving the bilateral basal ganglia, thalami, and subcortical regions, highlighted by red arrows.

The patient then returned to the ED in February 2024 with recurrent seizure-like activity, including convulsions, flailing of her extremities, disorientation, and sleepwalking. Per report, the patient was nonresponsive, hyperactive, and unable to follow commands when evaluated by EMS. Initial laboratory evaluation was notable for leukocytosis, with a WBC count of 13.1 × 10³/µL, and hypocalcemia, with a serum calcium level of 7.0 mg/dL, ionized calcium level of 0.70 mmol/L, low parathyroid hormone level of 2.3 pg/mL, phosphorus level of 5.1 mg/dL, magnesium level of 1.5 mg/dL, decreased 25-hydroxyvitamin D level of 14.6 ng/mL, and thyroid-stimulating hormone (TSH) level of 0.016 µIU/mL with a free T4 level of 1.68 ng/dL, suggestive of iatrogenic hyperthyroidism secondary to oral levothyroxine administration. A summary of the patient’s relevant endocrine and metabolic laboratory findings across hospital encounters is provided in Table 1. Given the complexity of her presentation, neurology and endocrinology were consulted.

**Table 1:** Relevant endocrine and metabolic laboratory findings across hospital encounters. AMA: Against medical advice; PTH: Parathyroid hormone; TSH: Thyroid-stimulating hormone.

Date	Intact PTH (pg/mL)	Serum calcium (mg/dL)	Serum ionized calcium (mmol/L)	Serum phosphorus (mg/dL)	Serum magnesium (mg/dL)	25-hydroxyvitamin D (ng/mL)	TSH (µIU/mL)	Free T4 (ng/dL)	Notes
Reference range	14.0-72.0	8.7-10.3	1.10-1.30	2.4-5.1	1.5-2.4	30.0-100.0	0.350-5.500	0.80-1.80	N/A
April 2023	0.1	7.9	-	5.8	2.4	76.1	591	0.36	Left AMA
February 2024	2.3	7.0	0.70	5.1	1.5	14.6	0.016	1.68	Discharged with decreased levothyroxine dosage; initiation of antiepileptics and oral calcium supplementation
March 2024	3.8	17.3	1.73	4.9	2.3	58.1	31.6	0.66	Calcium supplementation discontinued; left AMA

Electroencephalography demonstrated a modified burst-suppression pattern, with bursts of diffuse delta-range slowing separated by periods of suppression. Intermittent sharp waves were noted over the left frontotemporal region, suggestive of a possible epileptiform focus in this region; however, severe diffuse cerebral dysfunction, with medication effect as a possible contributing factor, was also considered. Lumbar puncture revealed 0 WBCs with normal protein and glucose levels, indicating no evidence of active infection or inflammation. CSF findings are summarized in Table 2. MRI of the brain showed no acute infarction but again demonstrated symmetric intracranial signal abnormalities corresponding to the known calcifications. Repeat MRI one week later showed no significant interval change (Figure 2).

**Table 2:** CSF findings from lumbar puncture performed during the February 2024 admission.

CSF parameter	Result	Reference range
Glucose	68 mg/dL	40-70 mg/dL
Protein	31.2 mg/dL	15-45 mg/dL
WBC count	0 cells/µL	0-5 cells/µL
RBC count	3 cells/µL	0 cells/µL
Appearance	Clear	Clear and colorless

**Figure 2:**
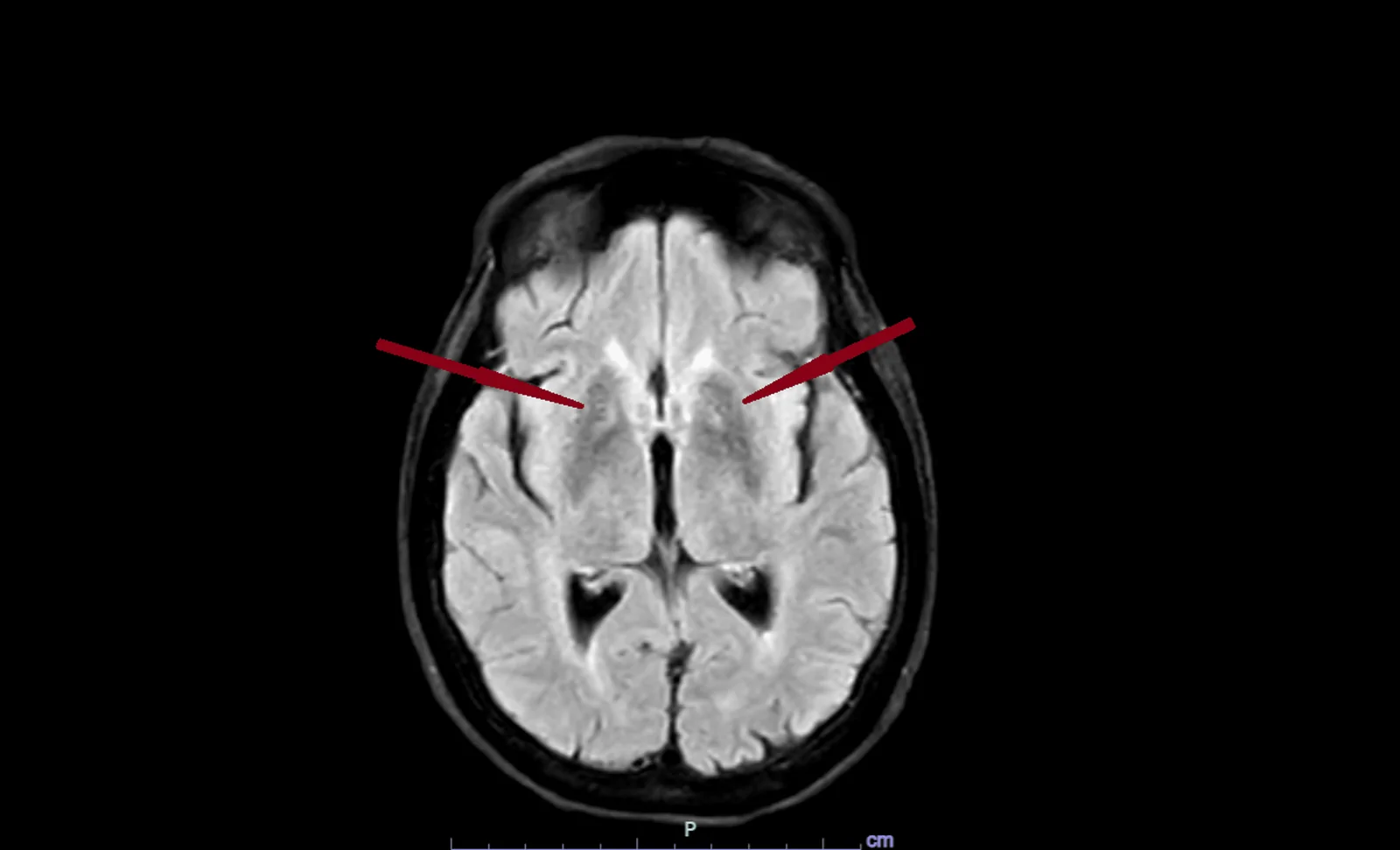
MRI of the brain demonstrating stable intracranial findings during the February 2024 admission. Axial T2-weighted fluid-attenuated inversion recovery (FLAIR) MRI of the brain demonstrates subtle, symmetric signal abnormalities involving the bilateral basal ganglia and deep gray matter regions, highlighted by red arrows, corresponding to known intracranial calcifications that are better visualized on computed tomography. No acute infarction was identified.

Following exclusion of infectious etiologies, antibiotics were discontinued. The patient was treated with brivaracetam 100 mg twice daily (BID) and valproic acid 500 mg every eight hours (Q8h), in addition to calcium supplementation with calcitriol 0.25 mcg BID and calcium carbonate 2500 mg three times daily (TID) for hypocalcemia. The patient was discharged with a plan to continue antiepileptic medications and calcium supplementation, decrease the levothyroxine dosage, maintain seizure precautions, and follow up closely with her primary care physician and neurology.

In March 2024, the patient again presented to the ED with weakness and dizziness. Laboratory evaluation revealed acute kidney injury, with a creatinine level of 1.5 mg/dL, increased from a baseline of 0.9-1.0 mg/dL, and an estimated glomerular filtration rate (eGFR) of 41 mL/min/1.73 m², decreased from a baseline of 67-70 mL/min/1.73 m², as well as severe hypercalcemia, with a serum calcium level of 17.3 mg/dL and ionized calcium level of 1.73 mmol/L, suspected to be secondary to oral calcium carbonate and calcitriol therapy. Laboratory evaluation also revealed a decreased parathyroid hormone level of 3.8 pg/mL, phosphorus level of 4.9 mg/dL, magnesium level of 2.3 mg/dL, 25-hydroxyvitamin D level of 58.1 ng/mL, and TSH level of 31.6 µIU/mL with a free T4 level of 0.66 ng/dL. The patient's hypothyroidism was suspected to be secondary to lack of adherence to her levothyroxine administration instructions, as she stated that she was taking levothyroxine with other medications. The patient's calcium replacement medications were discontinued, after which she reported symptomatic improvement. The patient once again elected to leave against medical advice prior to completion of her diagnostic workup. A timeline summarizing the patient’s diagnostic course, recurrent neurologic presentations, metabolic abnormalities, treatment changes, and discharges against medical advice is provided in Figure 3.

**Figure 3:**
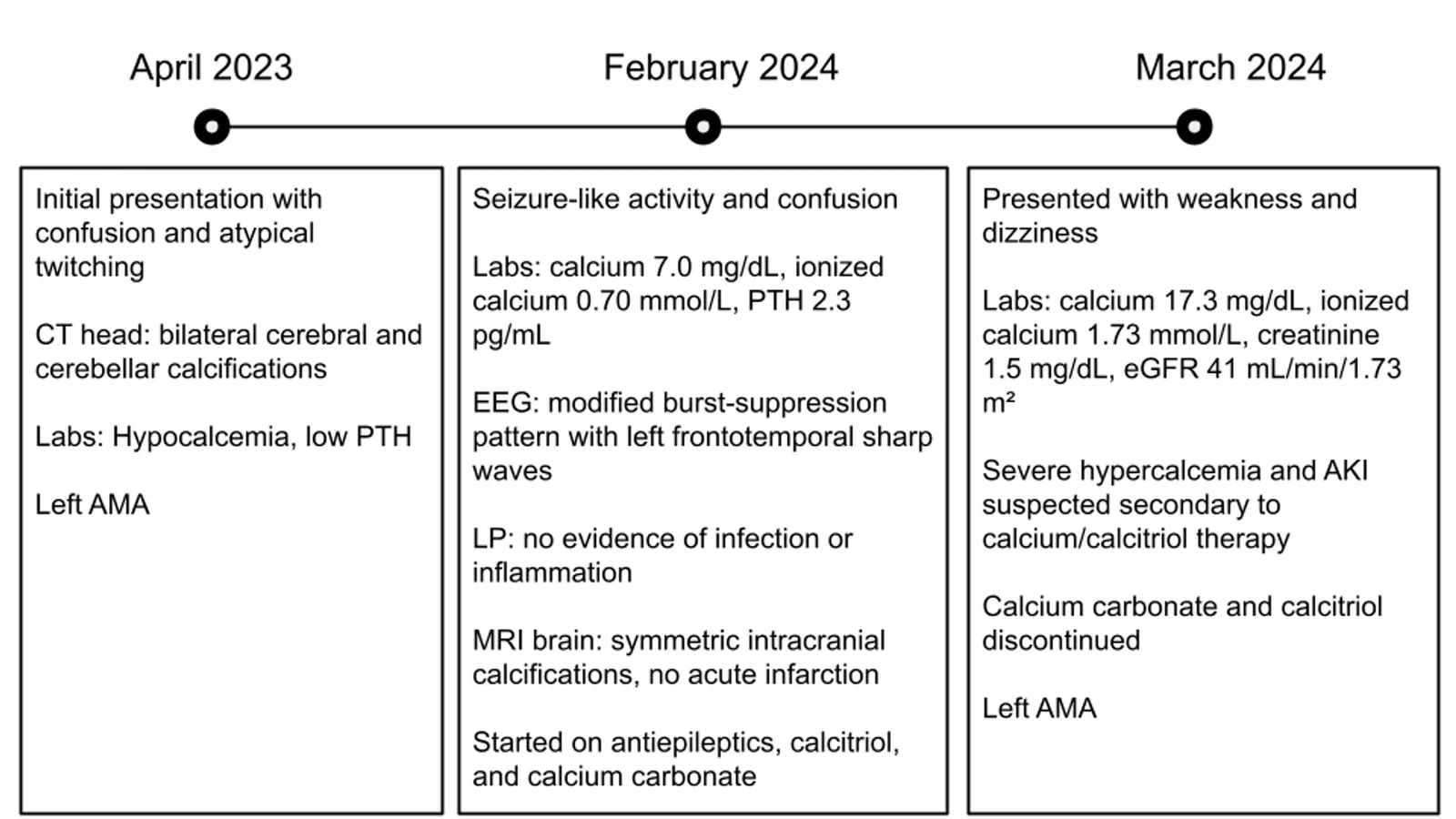
Timeline of the patient’s diagnostic course from April 2023 to March 2024. The timeline summarizes the patient’s recurrent presentations, initial identification of bilateral cerebral and cerebellar calcifications, hypocalcemia with low parathyroid hormone levels, recurrent seizure-like activity, treatment with antiepileptic therapy and calcium/calcitriol supplementation, subsequent severe hypercalcemia with acute kidney injury, and recurrent discharges against medical advice. AMA: Against medical advice; PTH: Parathyroid hormone; EEG: Electroencephalography; LP: Lumbar puncture; eGFR: Estimated glomerular filtration rate; AKI: Acute kidney injury. Note: The figure was created by the authors using Google Sheets without the use of generative AI.

## Discussion

This case highlights the diagnostic complexity of secondary Fahr syndrome in a patient with recurrent seizure-like activity, altered mental status, and extensive bilateral intracranial calcifications in the setting of chronic calcium-phosphate abnormalities. Although intracranial calcifications may be discovered incidentally, the patient’s recurrent neurologic symptoms and persistent metabolic derangements prompted further evaluation for secondary causes. Repeated neuroimaging demonstrated stable, extensive bilateral calcifications, while lumbar puncture and MRI showed no evidence of active infection, inflammation, or acute structural lesion. In combination with chronic hypocalcemia, hyperphosphatemia, and low parathyroid hormone levels, these findings supported a metabolic etiology most consistent with secondary Fahr syndrome related to hypoparathyroidism [1,4,9].

Disorders of calcium-phosphate metabolism are among the most important secondary causes of Fahr syndrome [4]. In hypoparathyroidism, low parathyroid hormone levels lead to impaired phosphate excretion and calcium regulation, producing the characteristic biochemical pattern of hypocalcemia and hyperphosphatemia. This imbalance may increase the calcium-phosphate product and promote ectopic deposition of calcium-containing complexes within intracranial small vessels and perivascular tissues [1,9]. Although the exact mechanism remains unclear, calcium deposition within vascular and perivascular structures has been proposed as one pathway linking intracranial calcifications to neurologic dysfunction [1,7]. A schematic representation of the proposed relationship between hypoparathyroidism, calcium-phosphate imbalance, intracranial calcium deposition, and neurologic manifestations is shown in Figure 4.

**Figure 4:**
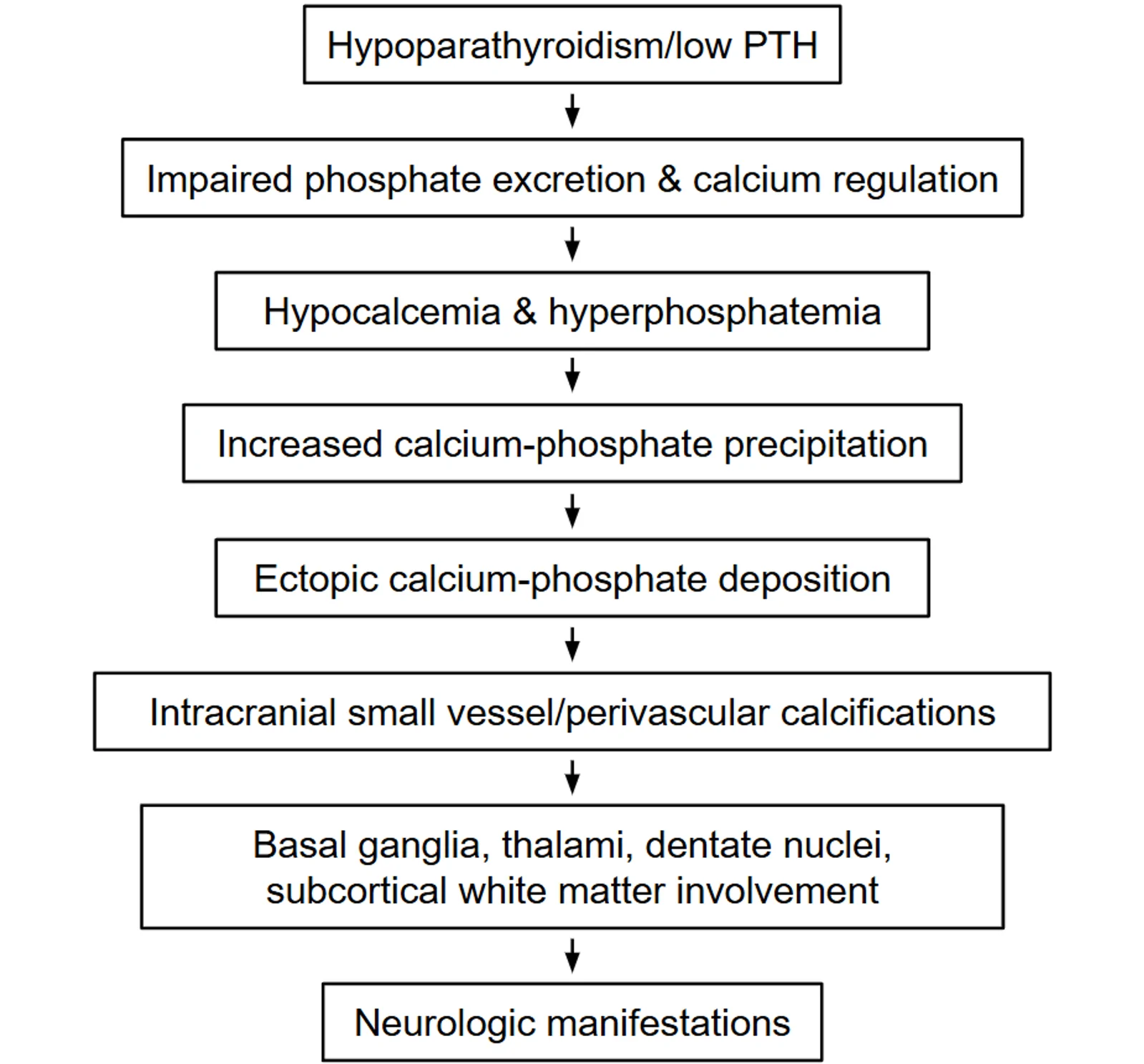
Proposed pathophysiologic relationship between hypoparathyroidism, calcium-phosphate imbalance, and secondary Fahr syndrome. Low parathyroid hormone (PTH) levels impair phosphate excretion and calcium regulation, leading to hypocalcemia, hyperphosphatemia, and calcium-phosphate imbalance. This biochemical environment may promote ectopic calcium-phosphate deposition within cerebral microvascular and perivascular structures, contributing to bilateral intracranial calcifications and associated neurologic manifestations [1,4,7,9]. Note: The figure was created by the authors using Google Slides without the use of generative AI.

Seizures and altered mental status are recognized neurologic manifestations of Fahr syndrome, although acute episodes may also be precipitated or worsened by metabolic derangements [1,4]. In this patient, recurrent hypocalcemia likely lowered the seizure threshold and contributed to her recurrent seizure-like activity and encephalopathy [10]. Her subsequent presentation with severe hypercalcemia after calcium carbonate and calcitriol therapy further illustrates the narrow therapeutic balance in patients with chronic hypocalcemia, hypoparathyroidism, and chronic kidney disease [11]. Because there is no definitive disease-modifying therapy for Fahr syndrome, treatment is largely symptomatic and directed toward controlling neurologic manifestations and correcting underlying metabolic abnormalities [12]. In this patient, acute management required neurologic stabilization with antiepileptic therapy and careful correction of metabolic abnormalities while avoiding overcorrection that could worsen renal function or produce symptomatic hypercalcemia.

Given the patient’s concurrent neurologic, endocrine, and renal abnormalities, close follow-up with neurology, endocrinology, nephrology, and primary care was necessary to monitor seizure control, calcium-phosphate balance, renal function, and thyroid replacement therapy. However, recurrent discharges against medical advice and inconsistent outpatient follow-up contributed to fragmented care and delayed diagnostic consolidation. Early recognition of secondary Fahr syndrome may help reduce unnecessary diagnostic testing, guide appropriate metabolic monitoring, and improve long-term care coordination in medically and socially complex patients.

## Conclusions

This case highlights the challenges of diagnosing and managing secondary Fahr syndrome in patients with significant medical comorbidities and complex social circumstances. Recurrent discharges against medical advice and inconsistent outpatient follow-up contributed to fragmented longitudinal care and delayed diagnostic clarification. Fahr syndrome should be considered in patients with recurrent neurologic symptoms and bilateral intracranial calcifications on neuroimaging, particularly in the setting of abnormalities in calcium-phosphate metabolism, such as hypocalcemia, hyperphosphatemia, and low parathyroid hormone levels suggestive of hypoparathyroidism. Although treatment is largely symptomatic and directed toward seizure control and correction of underlying metabolic abnormalities, this case underscores the need for careful monitoring to avoid both undertreatment and overcorrection of calcium abnormalities. Timely recognition may reduce unnecessary diagnostic testing, guide appropriate metabolic management, and improve long-term care coordination through a multidisciplinary, patient-centered approach.
